# Small-World Characteristics of EEG Patterns in Post-Anoxic Encephalopathy

**DOI:** 10.3389/fneur.2014.00097

**Published:** 2014-06-16

**Authors:** Martijn Beudel, Marleen C. Tjepkema-Cloostermans, Jochem H. Boersma, Michel J. A. M. van Putten

**Affiliations:** ^1^Department of Neurology and Clinical Neurophysiology, Medisch Spectrum Twente, Enschede, Netherlands; ^2^Department of Neurology, University Medical Centre Groningen, Groningen, Netherlands; ^3^Department of Clinical Neurophysiology, MIRA Institute for Biomedical Technology and Technical Medicine, University of Twente, Enschede, Netherlands

**Keywords:** small-world network, continuous EEG, post-anoxic encephalopathy, prognosis, resuscitation

## Abstract

Post-anoxic encephalopathy (PAE) has a heterogenous outcome which is difficult to predict. At present, it is possible to predict poor outcome using somatosensory evoked potentials in only a minority of the patients at an early stage. In addition, it remains difficult to predict good outcome at an early stage. Network architecture, as can be quantified with continuous electroencephalography (cEEG), may serve as a candidate measure for predicting neurological outcome. Here, we explore whether cEEG monitoring can be used to detect the integrity of neural network architecture in patients with PAE after cardiac arrest. From 56 patients with PAE treated with mild therapeutic hypothermia, 19-channel cEEG data were recorded starting as soon as possible after cardiac arrest. Adjacency matrices of shared frequencies between 1 and 25 Hz of the EEG channels were obtained using Fourier transformations. Number of network nodes and connections, clustering coefficient (*C*), average path length (*L*), and small-world index (SWI) were derived. Outcome was quantified by the best cerebral performance category (CPC)-score within 6 months. Compared to non-survivors, survivors showed significantly more nodes and connections. *L* was significantly higher and *C* and SWI were significantly lower in the survivor group than in the non-survivor group. The number of nodes, connections, and the *L* were negatively correlated with the CPC-score. *C* and SWI correlated positively with the CPC-score. The combination of number of nodes, connections, *C*, and *L* showed the most significant difference and correlation between survivors and non-survivors and CPC-score. Our data might implicate that non-survivors have insufficient distribution and differentiation of neural activity for regaining normal brain function. These network differences, already present during hypothermia, might be further developed as early prognostic markers. The predictive values are however still inferior to current practice parameters.

## Introduction

Post-anoxic encephalopathy (PAE) has a heterogenous outcome which is difficult to predict. Good outcome or outcome with moderate disabilities is present in ~50% of the patients who make it alive to the hospital after resuscitation (1). At present, prediction of either good or poor outcome is limited. The main drawback of parameters for early outcome prediction based on motor score, brainstem reflexes, and somatosensory evoked potentials (SSEPs) (2, 3) is their limited sensitivity or specificity. Recently, the value of these items was re-established in patients treated with mild therapeutic hypothermia (MTH) (1). Since early predictors of prognosis can both reduce the costs of intensive care unit (ICU) stay in case of poor prognosis and give more accurate information to the relatives of patients, more reliable predictors are of great material and immaterial value. Given its non-invasive character in combination with its real time reflection of cerebral state, EEG features may be a potential additional predictor in PAE.

At present, the role of the EEG in prediction of neurological outcome in patients with PAE is a subject of active inquiry. In a recent study, the absence of EEG reactivity had a sensitivity of 75% and a specificity of 100% for poor outcome (4). However, another recent study on EEG reactivity between 1 and 3 days after the anoxic event had a specificity of only 94% for predicting poor outcome (5). Furthermore, in the literature burst suppression on EEG is reported to have a specificity between 85 and 100% (4, 6, 7) and post-anoxic status epilepticus on EEG had a specificity of 98% for poor outcome (6). In a recent study, low-voltage and iso-electric EEG patterns were shown to be invariably associated with poor neurological outcome 24 h after the event in patients treated with MTH and sedation with a sensitivity of 40% and a specificity of 100%, while bilaterally absent cortical SSEP had a sensitivity of only 24% after 24 h in this study (7).

Currently, some drawbacks exist in the use of EEG in PAE prognostication. In general, its inter-observer agreement is only moderate (8, 9) and interpretation highly depends on qualified personnel. For these reasons, additional automatized and quantitative EEG analyses, which give a real time reflection of cerebral state, are of potential value in prediction of outcome in comatose patients with PAE (10). EEG derived neural network properties might reflect this cerebral state. In the present study, we aimed to use quantitative EEG network analysis for outcome prediction in patients with PAE and analyze EEG network differences between PAE survivors and non-survivors. To understand the relation between neural network properties and current clinical parameters, we also compared EEG neural network properties with SSEP data and visually interpreted EEG data.

Graph theory has become a powerful tool to quantify network properties (11–13). Graphs are representations of a set of objects where some pairs of these objects are connected by links. Graphs can be characterized by a clustering coefficient (*C*), indicating the degree of local clustering of network elements, and a average path length (*L*), defined as the average number of steps along the shortest paths for all possible pairs of network elements (14). So-called “small-world” network properties (11), network properties defined by both a high local clustering between adjacent nodes (*C*) and a short average path length (*L*) between all network nodes, might reflect optimal (cerebral) processing (15). Recent fMRI and EEG studies indicate a role for small-world based cerebral processing related to optimal cerebral performance (16), for review see Ref. (17). Next to this, in patients with temporal lobe epilepsy, small-world properties of cortical thickness based networks were impaired (18). However, in hippocampal modeling small-world network properties resulted in seizure vulnerability (19). Using resting state fMRI, altered small-world network properties were seen in an outpatient population with hepatic encephalopathy (20). Given the different applied methods (e.g., EEG or MRI) and outcome parameters (e.g., anatomical or physiological) it is difficult to compare the various studies on small-world network properties and brain function. Since PAE generally results in a decreased level of consciousness, both due to anoxic damage and sedation, small-world architecture might be disrupted. For this reason, our hypothesis was that the average path length would be higher and the clusterings coefficient would be lower in the patients with more severe anoxic damage. Given the finding that large-scale small-world properties of cerebral processing are preserved during anesthesia (21, 22), these proposed disruptions of small-world architecture might be a consequence of the anoxic damage and not of the anesthesia.

One way of studying small-world properties by using EEG is by looking at shared frequencies between the different electrodes presumably reflecting synchronization between groups of neurons (23). Based on the conception that if groups of neurons synchronize functional interactions are present (24), these shared frequencies might reflect a neural network from which small-world characteristics (14) can be derived. These small-world characteristics of PAE patients were analyzed using continuous electroencephalography (cEEG) registration during ICU admission and were correlated with the best achieved cerebral performance category (CPC) score within 6 months.

## Materials and Methods

### Subjects

Sixty PAE patients who survived, but remained comatose, after cardiopulmonary resuscitation and were treated with TH were included in the study. Outcome prediction based on SSEP and visual analysis of the EEG data was recently published (7). The local medical ethics committee waived the need for informed consent for EEG monitoring during ICU stay. However, for additional electrophysiological and clinical evaluation after discharge from the ICU, local institutional review board approval and written informed consents were obtained. Exclusion criteria were age <18 years, other concurrent neurological pathology or a known history of severe neurological disorders, and brain surgery or neuro-trauma. MTH of 33°C was induced and maintained for 24 h. Further patient characteristics can be found in Ref. (7).

### Data acquisition

EEG recordings were started as soon as possible after the patients’ arrival on the ICU and continued up to 5 days or until discharge from the ICU or until patients lived. Twenty-one EEG electrodes were attached to the skull according to the international 10–20 system. Recordings were made using a Neurocenter EEG recording system (Clinical Science Systems, Voorschoten, The Netherlands) using a sample frequency of 256 Hz. EEG signals were recorded using an average reference and band-pass filtered from 1 to 100 Hz.

### Outcome assessment

The primary outcome measure was the best score within 6 months on the five point CPC-score (25).

### Data analysis

In the first 24 h after the event, EEG intervals of 5 min (300 s) were selected for each hour of registration. At 48 h after the event, intervals were selected every 2 h. Intervals of insufficient recording quality (e.g., due to artifacts) were excluded. All analyses were performed using Matlab 2011a (The MathWorks 2011).

Dominant frequencies present in the spectrum of the 19 EEG channels of the EEG intervals were detected by Fast Fourier Transformations (FFT). For all 300 s of the interval, a frequency spectrum with a resolution of 0.5 Hz ranging from 1 to 25 Hz was obtained. A Hann-window of 2 s was used resulting in an overlap of 50%. Frequencies were considered significant when (a) the amplitude of a frequency’s Fourier coefficient was at least 5% of the maximum of that frequency in the surrounding 4 s, (b) the value of the Fourier coefficient was at least 50% of the coefficient with the maximum amplitude within the Hann-window, and (c) the value was larger than the neighboring four Fourier coefficients.

For every 300-s, the frequencies present in each channel were compared with the frequencies present in the other 18 channels. In case one or more similar frequencies were present in two channels, the channels were presumed to have a connection. The absence or presence of all possible connections (19 × 19) was put into a dichotomous (ones and zeros), adjacency matrix for each second. In case no detectable oscillation between 1 and 25 Hz was present in the signal of an electrode, the matrix element of the electrode matching itself was zero. In case one or more oscillations were detected the matrix element was one. The network characteristics (see next section) of all seconds of each interval were averaged resulting in one value per interval of 300 s.

From the adjacency matrices, the network size (number of 19 electrodes present in a network) and the number of connections between the 19 electrodes were derived. *C* and *L* were derived from the adjacency matrices using the “brain connectivity toolbox” (26). *C* was obtained by inferring whether the connections of each electrode were also mutually connected. For each electrode, *C* was expressed as a fraction of the maximum number of possible connections between its connections, so-called “neighboring neighbors.” For example, when an electrode was connected with three other electrodes, three possible connections between its connections could maximally be present. The values of the individual electrodes forming the network were subsequently averaged. *L* was obtained by calculating the average shortest path length from each electrode to each other electrode in the network. Given the dependency of *C* and *L* on network size and number of connections, *C* and *L* were expressed as a fraction of *C* and *L* of random graphs with a similar network size and number of connections. Next to *C* and *L*, the small-world index (SWI = *C*/*L*) was obtained for every subject for every epoch. The larger the SWI value is, the more small-world the network is. For further analyses, the values of the epochs of each hour were averaged over the intervals between 0 and 24, 0 and 48, and 0 and 72 h. In case not every epoch was available (e.g., when the registration stopped at 40 h), the average value for the registered epochs was used.

For the episodes between 0 and 24, 0 and 48 and 0 and 72 h, comparisons of network size, number of connections, *C, L*, and SWI between non-survivors (CPC = 5) and survivors (CPC < 5) were made using a two-sample *t*-test. Next to this, a combination of network size, number of connections, *C*, and *L* was analyzed non-parametrically using the Wilcoxon rank sum test based on the sum ranks of network size, number of connections, *C*, and (inverse) *L*. Furthermore, correlation coefficients for the aforementioned characteristics and the CPC-scores were obtained. Receiver operating characteristic (ROC) analyses were conducted to explore the sensitivity and specificity profile of the aforementioned network characteristics.

These aforementioned network characteristics were also compared with SSEP and EEG patterns after 24 h [for description of the data acquisition see Ref. (7)]. The network characteristics were divided into a group with bilateral absent SSEP or uni- or bilateral present SSEP. The resulting groups were compared using two-sample *t*-tests. The same categories for EEG visual interpretation as in Ref. (7) were used. In line with the previous publication, the unfavorable EEG patterns, iso-electric and low-voltage, were combined. Network characteristics of the unfavorable iso-electric and low-voltage EEGs were compared with the more favorable EEGs of diffuse slowing using two-sample *t*-tests.

## Results

Data of 56 of the 60 included patients (mean age 68 ± 12, 38 male) were used. Four patients were excluded in a later stage, two because of intracerebral hemorrhage, one because of technical problems during the EEG registration, and one because of death during the first hour of registration. None of the remaining 56 patients was lost during follow-up.

### Neurological outcome

Within 6 months, 27 of the 56 eligible subjects died (CPC = 5). The other 29 had a outcome varying from normal cerebral function (CPC = 1, *n* = 19) to mild (CPC = 2, *n* = 8), modest (CPC = 3, *n* = 1),or severe (CPC = 4, *n* = 1) cerebral dysfunction.

### Matrices of characteristic EEG patterns

**Figure 1:**
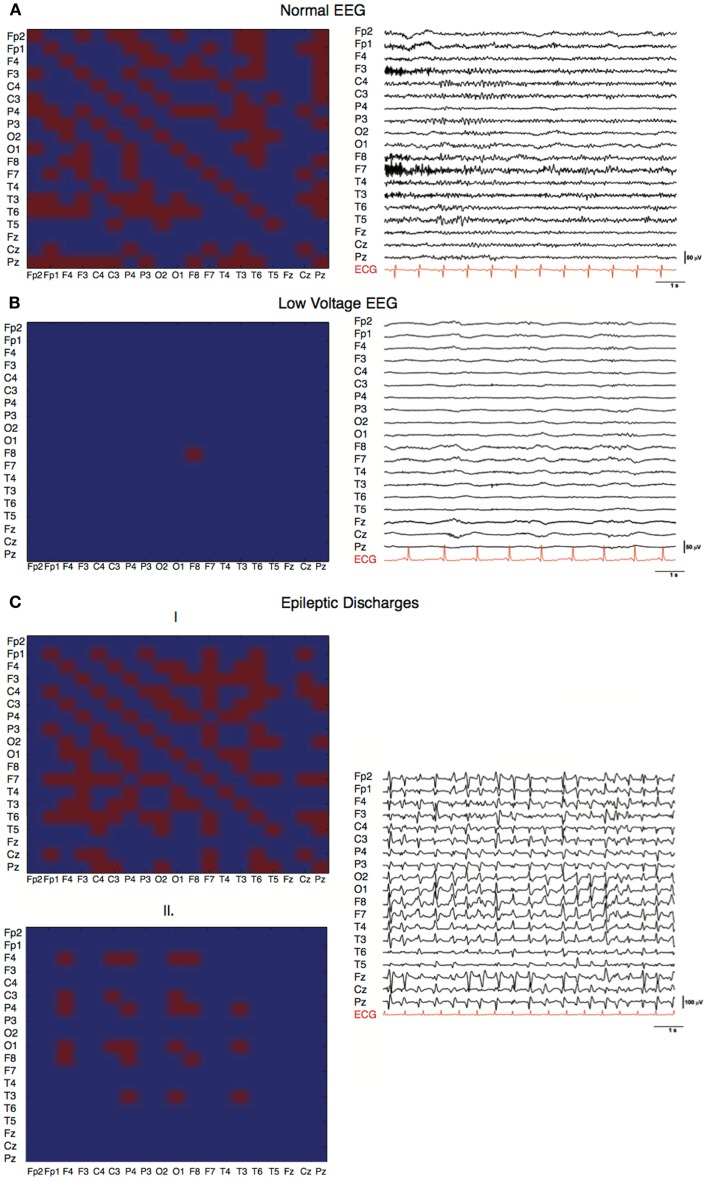
**Characteristic EEGs and their network configurations**. Horizontal and vertical axes show the different EEG electrodes. Red squares represent connections, blue squares represent absent connections. **(A)** Adjacency matrix of a PAE patient with a good EEG showing involvement of 160 of the possible 361 (19 × 19) connections and involvement of 18 of the 19 electrodes in the network (*C* = 0.81, *L* = 1.77, SWI = 0.45). **(B)** Adjacency matrix of a PAE patient with a low-voltage EEG pattern (*C* = 0, *L* = 1, SWI = 0). Only one electrode had sufficient power to produce a detectable EEG wave. No network was present. **(C)** Adjacency matrices of a PAE patient showing generalized epileptic discharges with varying network size and number of connections within a 5-min epoch, (1) *C* = 0.32, *L* = 1.33, SWI = 0.24; (2) *C* = 0.86, *L* = 1.76, SWI = 0.48.

Three characteristic EEG patterns normal, generalized periodic discharges, and low-voltage were analyzed at the individual level. The common denominator of the different network characteristics of a normal EEG (i.e., continuous with non-encephalopathic background rhythm) within a 5-min interval (Figure 1A) was an involvement of almost all electrodes with many, although fluctuating, connections. Compared with the adjacency matrix of the normal EEG, the adjacency matrices of the EEG with generalized periodic discharges showed a pattern with more variation within a 5-min interval with more pronounced discharges (Figure 1C1) against a low background (Figure 1C2). The low-voltage EEG (Figure 1B) showed no network activity and only one individual oscillation was detected by a single electrode.

### Network size

**Figure 2:**
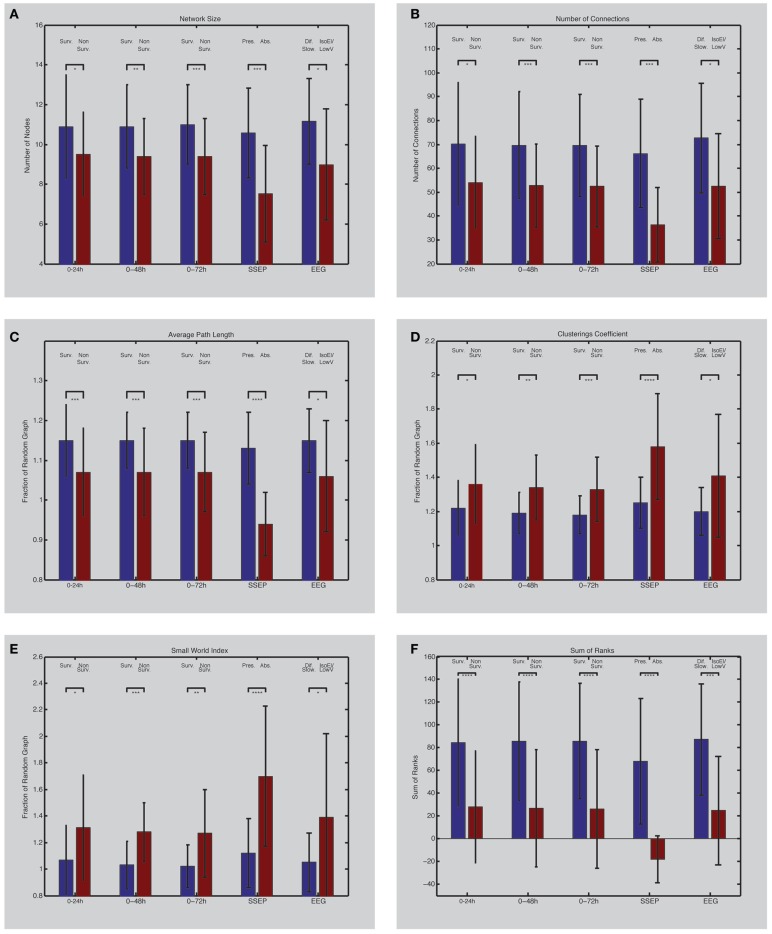
**(Left) EEG network characteristics of PAE survivors and non-survivors**. Depicted are differences between survivors and non-survivors (first three bar couples) and their values between 0 and 24 h (1), 0 and 48 h (2), and 0 and 72 h (3) after the arrest (forth bar couple); differences between patients with bilateral absent SSEP and uni- or bilateral present SSEP (fifth bar couple); differences between patients with iso-electric or low-voltage EEG and diffuse slowing on EEG. Network size **(A)** is defined by the number of electrodes present in the networks. Number of connections in the network is shown in **(B)**. Average path length **(C)** and clustering coefficient **(D)** are expressed as a fraction compared to a random network with similar size and connections. The small-world index **(E)** was obtained by dividing the clustering coefficient with the average path length. The sum of ranks **(F)** was obtained by the non-parametrical analysis of the ranked parameters **(A–D)**. Asterisks indicate the level of significance and error bars represent standard deviations. *p < 0.05, **p < 0.01, ***p < 0.005, ****p ≤ 0.001. Surv., survivors; Non-Surv., non-survivors; Pres., uni- or bilateral present SSEP; Abs., absent SSEP; Dif. Slow., EEG with diffuse slowing; IsoEl/LowV, iso-electric of low-voltage EEG.

Comparing the network size of the non-survivors with the survivors revealed a consistent larger network size over the entire registration period in the survivor group. Comparing the average network size of the first 24 h between the survivor and non-survivor group showed a significant larger network in the survivor group (Table 1; Figure 2A). This difference persisted after 48 (0–48 h) and 72 h (0–72 h) (Table 1; Figure 2A). A significant correlation was present between the CPC-score and the average network size of the first 24 h (*p* = 0.02, ρ = −0.29). For the first 48 h, this correlation also turned out to be more robust (*p* = 0.005, ρ = −0.36), and for the first 72 h even more robust (*p* = 0.003, ρ = −0.38).

**Table 1:** **Composition of patient population after 24, 48, and 72 h and their network characteristics including standard deviations and *p* values**.

	Interval (h)	Non-survivors	Survivors	*p* Value
Number		27	29	
Remaining subjects	24	22	27	
	48	13	22	
	72	12	14	
Network size	0–24	9.5 ± 2.1	10.9 ± 2.6	0.04
	0–48	9.4 ± 1.9	10.9 ± 2.1	0.007
	0–72	9.4 ± 1.9	11.0 ± 2.0	0.004
Number of connections	0–24	54.1 ± 19.3	70.1 ± 25.7	0.02
	0–48	52.7 ± 17.4	69.6 ± 22.4	0.003
	0–72	52.4 ± 16.9	69.5 ± 21.4	0.002
Average path length	0–24	1.07 ± 0.11	1.15 ± 0.09	0.005
	0–48	1.07 ± 0.1	1.15 ± 0.07	0.002
	0–72	1.07 ± 0.11	1.15 ± 0.07	0.0009
Clustering coefficient	0–24	1.36 ± 0.23	1.22 ± 0.16	0.02
	0–48	1.34 ± 0.19	1.19 ± 0.12	0.007
	0–72	1.18 ± 0.11	1.33 ± 0.19	0.001
Small-world index	0–24	1.31 ± 0.4	1.07 ± 0.26	0.02
	0–48	1.28 ± 0.22	1.03 ± 0.18	0.002
	0–72	1.27 ± 0.33	1.02 ± 0.16	0.009
Sum of ranks	0–24	28.1 ± 49.1	84.2 ± 55.9	0.0003
	0–48	26.7 ± 51.5	85.6 ± 52.2	0.00009
	0–72	26.3 ± 52.2	85.9 ± 50.7	0.00007

### Number of connections

The number of connections also turned out to be higher in the survivor group over the entire registration relative to the non-survivor group. Comparing the average number of connections of the first 24 h between the survivor and non-survivor group showed a significant increased number of connections in the survivor group (Table 1; Figure 2B). This difference persisted after 48 and 72 h (Table 1; Figure 2B). Average network size of the first 24 h correlated significantly with CPC-score (*p* = 0.006, ρ = −0.35). This correlation strengthened after 48 (*p* = 0.001, ρ = −0.41) and 72 h (*p* = 0.001, ρ = −0.43).

### Average path length

After correction for network size, the relative average path length (*L*) was higher in the survivor compared to the non-survivor group during the entire registration. In the first 24 h, *L* was significantly higher in the survivor group compared to the non-survivor group (Table 1; Figure 2C). After 48 and 72 h, this difference persisted (Table 1; Figure 2C). The *L* also significantly correlated with CPC-score (*p* = 0.004, ρ = −0.37 after 24 h; *p* = 0.001, ρ = −0.41 after 48 h; and *p* = 0.001, ρ = −0.42 after 72 h).

### Clustering coefficient

The relative clustering coefficient (*C*) was lower in the survivor group compared to the non-survivor group during the entire registration. Comparing the relative *C* of the first 24 h between the survivor and non-survivor group showed a significant lower *C* in the survivor group (Table 1; Figure 2D). After 48 h, this statistical difference increased and further increased after 72 h (Table 1; Figure 2D). *C* correlated significantly with the CPC-score (*p* = 0.009, ρ = 0.34 after 24 h; *p* = 0.001, ρ = 0.42 after 48 h; and *p* = 0.0009, ρ = 0.43 after 72 h).

### Small-world index

The SWI was lower for the survivor group compared to the non-survivor group during the entire registration. Comparing the SWI of the first 24 h between the survivor and non-survivor group showed a significantly lower SWI in the survivor group (Table 1; Figure 2E). After 48 and 72 h, this difference persisted (Table 1; Figure 2E). The SWI also correlated significantly with the CPC-score (*p* = 0.01, ρ = 0.33 after 24 h; *p* = 0.001, ρ = 0.40 after 48 h; and *p* = 0.001, ρ = 0.41 after 72 h).

### Sum of ranks

The combination of the ranks of the variables, network size, number of connections, relative average path length, and relative clustering coefficient showed the biggest statistical difference between the survivors and non-survivors (Table 1; Figure 2F). After 48 and 72 h, these differences persisted (Table 1; Figure 2F). This combination showed also a significant correlation with the CPC-score (*p* = 0.0001, ρ = −0.49, after 24 h; *p* = 0.0001, ρ = −0.51, after 48 and 72 h).

### ROC analyses

Although significant differences were present for all the network parameters at the group level, the network parameters lacked sufficient sensitivity to discriminate non-survival from survival at a specificity level of 100%.

### Comparison with SSEP

From the 56 patients, 7 had a bilateral absent SSEP. All of them did not survive. After 24 h, all the previously described network characteristics differed significantly between the 7 patients with a bilateral absent SSEP and the 49 patients with uni- or bilateral present SSEP (Table 2A; Figures 2A–F). The direction of the differences was also similar as in the comparison using the CPC-scores; patients with bilateral absent SEPP showed smaller networks, less connections, and more small-world topology relative to patients with uni- or bilateral present SSEP.

**Table 2:** **(A) Difference between network characteristics of patients with bilateral absent SSEP and uni- or bilateral present SSEP including standard deviations and *p* values and (B) difference between network characteristics of patients with iso-electric or low-voltage EEG including standard deviations and *p* values**.

**(A)**			
	Bilateral absent SSEP	Bi- or unilateral present SSEP	*p* Value
Number	7	49	
Network size	7.52 ± 2.43	10.57 ± 2.25	0.0017
Number of connections	36.36 ± 15.7	66.17 ± 22.7	0.0015
Average path length	0.94 ± 0.08	1.13 ± 0.09	0.00001
Clusterings coefficient	1.58 ± 0.31	1.25 ± 0.15	0.00004
Small-world index	1.70 ± 0.53	1.12 ± 0.26	0.00001
Sum of ranks	-18.28 ± 20.67	68.00 ± 55.19	0.0001

**(B)**			

	**Iso-electric/low-voltage**	**Diffuse slowing**	***p* Value**

Number	8	26	
Network size	8.99 ± 2.79	11.15 ± 2.15	0.02
Number of connections	52.46 ± 22.03	72.69 ± 22.93	0.03
Average path length	1.06 ± 0.14	1.15 ± 0.08	0.03
Clusterings coefficient	1.41 ± 0.36	1.20 ± 0.14	0.02
Small-world index	1.39 ± 0.63	1.05 ± 0.22	0.02
Sum of ranks	24.7 ± 47.73	87.3 ± 48.7	0.003

### Comparison with visual EEG interpretation

After 24 h, 46 of the 56 patients still had continuous EEG registration. Two had an iso-electric EEG, 6 had a low-voltage EEG, 12 had an burst suppression EEG, and 26 had an EEG with diffuse slowing [see Ref. (7)]. All network characteristics differed significantly between the iso-electric and low-voltage group and the diffuse slowing group (Table 2B; Figures 2A–F). The direction of the differences was also similar as in the comparison using the CPC-scores and SSEP; patients with iso-electric of low-voltage EEG showed smaller networks, less connections, and more small-world topology relative to patients with diffuse slowing.

## Discussion

In this study, we investigated whether cEEG monitoring can be used for evaluating the integrity of neural network architecture in PAE patients and differentiate between PAE survivors and non-survivors. Graph analysis revealed consistent differences between survivors and non-survivors of PAE already present during the MTH period. At group level, PAE patients with the best neurological outcome had the most widespread and most densely connected networks. Corrected for network size and number of connections, the PAE patients with the worst neurological outcome had the network characteristics most resembling small-world networks. These differences might indicate that the PAE patients with the worst neurological outcome lack sufficiently widespread and connected networks and lack sufficiently differentiated connectivity in the remaining network. However, sensitivity and specificity are inferior to current prognostic parameters. Further optimization of the graph analyses in the temporal (real time analyses) and spatial (multi-channel analyses) domain might develop graph analyses as a tool for early prognostication in PAE.

### Network disruptions in anoxic coma

The decreased network size and the decreased number of connections in these networks in PAE patients with a poor outcome are the end result of failing neural communication. The most crucial aspect of this neural communication is the synaptic transmission. Synaptic transmission is a metabolically demanding process which is blocked at first in anoxia (27). In anoxia, especially glutaminergic, synaptic failure (28) might lead to functional disconnection and consequently network failure. However, this synaptic failure is putatively reversible (28). When anoxia is more severe, not only functional but also structural changes occur leading to irreversible transmission failure (29).

In global anoxia, the entire brain is withdrawn from oxygen. However, local differences in vulnerability to anoxia exist (30). This is for example the case for the metabolically active hippocampus (30). Whether such higher vulnerability also exists for longer, metabolically more demanding (31), cortico-cortical connections, and establishing large neural networks is not known. Hypothetically, since the metabolic cost of spike transmission scales up linearly with connection length (31), rapid disruption of axon–glial connections (32) after anoxia might preferentially result in the breakdown of these connections.

The networks of the PAE patients with a poor outcome and a more small-world like organization might indicate that the cortical networks of these patients are both under-dimensioned (smaller) and lacking complexity (less differentiated). This organization might reflect a disturbed balance between neuronal integration and differentiation (33), in which small cortical areas are relatively hyper-connected.

No studies on small-world characteristics of post-anoxic patients have been conducted so far. Our finding that differences in small-world characteristics exist between survivors and non-survivors and correlate with outcome also supports the functional difference between coma induced by anesthesia (21, 22) and anoxia. Further analyses in the temporal domain could elucidate possible differences in neural synchronization and de-synchronization (34, 35) between those patients who regain consciousness and those who do not.

### Sedation

Differences in sedation levels may have influenced the EEG patterns. However, no significant difference in sedation level between the group with good neurological outcome and poor neurological outcome was found [see Ref. (7)]. Furthermore, it is unlikely that the most severe EEG patterns (iso-electric and low-voltage) were caused by the use of propofol, fentanyl, or remifentanil in the doses used, as the EEG is not suppressed at these doses, and typically only shows moderate slowing (36).

### Methodology

With our approach, we cannot exclude that a common driver or volume conduction (37) is responsible for similar frequencies recorded from different electrode positions. Therefore, use of techniques that remove these potential contributions to spurious synchrony (38) would likely better reflect actual functional connections (38–40). However, the applied method appears still a useful metric that significantly differed in these patients. Since the same method was applied for every subject, independent of outcome, no systematic bias occurred. Even then, one must be cautious with the physiological interpretation of the various metrics found, however, which is a general concern in these approaches. Indeed the relation between functional connectivity and physiological reality is not trivial, in particular when metrics are derived from scalp EEG recordings (40, 41). However, the applied method does result in significant differences in the metrics between the patients studied, with potential pragmatic applicability in a clinical setting.

Besides this, graph analysis is a uniform way of looking at EEG patterns without *a priori* considering conventional aspects like iso-electric patterns, low-voltage patterns, diffuse slowing, burst suppression patterns, generalized periodic discharges, or epileptiform discharges (7). In case of an iso-electric EEG, no neural (network) activity is present, this is also most often the case in low-voltage EEGs. In one patient with a low-voltage EEG only one network element was present (Figure 1C). On the contrary, when EEGs were almost normal the most connections and nodes were present (Figure 1A). In other patterns, burst suppression and generalized epileptic discharges (Figures 1C1 and 2), different network characteristics were present varying from virtually absent networks to larger more connected networks. This is due to the fact that these patterns were not continuous and network characteristics varied over time. The critical pattern characteristics averaged out when conducting our algorithm for episodes of 5 min. Further analyses on different time scales with different spectra might elucidate deviant network characteristics of these typical EEG patterns. However, this is beyond the scope of the current paper. The additional correlation analyses show that, although CPC-scores 2–4 were relatively underrepresented, the mentioned differences are gradual and not dichotomous. This makes it more difficult to apply dichotomous decision protocols.

The networks characteristics average path length and clustering coefficient, and consequently the SWI, that were obtained in our data (independent of outcome) did not show the magnitudes that are associated with small-worldness (SWI = 2) (14). For this reason one should speak of a tendency toward small-worldness. This does not mean that there are no significant differences between the small-world parameters and between the two groups but that the data more tend toward a more random order or regular order (11).

## Conclusion

In summary, EEG network analyses revealed significant differences between PAE survivors and non-survivors. Non-survivors showed smaller, less connected networks that were configured more toward small-world architecture. These network characteristics were also correlated with CPC-score and were already present during hypothermia. Further development of EEG network analyses can result in an instantaneous and easy to use prognostic tool for PAE patients.

## Conflict of Interest Statement

The authors declare that the research was conducted in the absence of any commercial or financial relationships that could be construed as a potential conflict of interest.

## References

[1] Bouwes A Binnekade JM Kuiper MA Bosch FH Zandstra DF Toornvliet AC Prognosis of coma after therapeutic hypothermia: a prospective cohort study. Ann Neurol (2012) 71:206–12 10.1002/ana.22632 22367993

[2] Wijdicks EF Hijdra A Young GB Bassetti CL Wiebe S Quality Standards Subcommittee of the American Academy of Neurology Practice parameter: prediction of outcome in comatose survivors after cardiopulmonary resuscitation (an evidence-based review): report of the Quality Standards Subcommittee of the American Academy of Neurology. Neurology (2006) 67:203–10 10.1212/01.wnl.0000227183.21314.cd 16864809

[3] Zandbergen EG Hijdra A Koelman JH Hart AA Vos PE Verbeek MM Prediction of poor outcome within the first 3 days of postanoxic coma. Neurology (2006) 66:62–8 10.1212/01.wnl.0000191308.22233.88 16401847

[4] Rossetti AO Urbano LA Delodder F Kaplan PW Oddo M Prognostic value of continuous EEG monitoring during therapeutic hypothermia after cardiac arrest. Crit Care (2010) 14:R173 10.1186/cc9276 20920227 PMC3219275

[5] Thenayan EA Savard M Sharpe MD Norton L Young B Electroencephalogram for prognosis after cardiac arrest. J Crit Care (2010) 25:300–4 10.1016/j.jcrc.2009.06.049 19781908

[6] Rundgren M Westhall E Cronberg T Rosén I Friberg H Continuous amplitude-integrated electroencephalogram predicts outcome in hypothermia-treated cardiac arrest patients. Crit Care Med (2010) 38:1838–44 10.1097/CCM.0b013e3181eaa1e7 20562694

[7] Cloostermans MC van Meulen FB Eertman CJ Hom HW van Putten MJ Continuous electroencephalography monitoring for early prediction of neurological outcome in postanoxic patients after cardiac arrest: a prospective cohort study. Crit Care Med (2012) 40:2867–75 10.1097/CCM.0b013e31825b94f0 22824933

[8] Beniczky S Aurlien H Brøgger JC Fuglsang-Frederiksen A Martins-da-Silva A Trinka E Standardized computer-based organized reporting of EEG: SCORE. Epilepsia (2013) 54(6):1112–24 10.1111/epi.12135 23506075 PMC3759702

[9] Gerber PA Chapman KE Chung SS Drees C Maganti RK Ng Y-T Interobserver agreement in the interpretation of EEG patterns in critically ill adults. J Clin Neurophysiol (2008) 25:241–9 10.1097/WNP.0b013e318182ed67 18791475

[10] Tjepkema-Cloostermans MC van Meulen FB Meinsma G van Putten MJ A cerebral recovery index (CRI) for early prognosis in patients after cardiac arrest. Crit Care (2013) 17:R252 10.1186/cc13078 24148747 PMC4056571

[11] Watts DJ Strogatz SH Collective dynamics of “small-world” networks. Nature (1998) 393:440–2 10.1038/30918 9623998

[12] Travers J Milgram S An experimental study of the small world problem. Sociometry (1969) 32(4):425–43 10.2307/2786545

[13] Barabási A-L Linked. New York: Basic Books (2002).

[14] Sporns O Small-world connectivity, motif composition, and complexity of fractal neuronal connections. Biosystems (2006) 85:55–64 10.1016/j.biosystems.2006.02.008 16757100

[15] Du H Wu X Zhuang J Small-World Optimization Algorithm for Function Optimization, in Advances in Natural Computation, eds Licheng J. Lipo W. Xinbo G. Jing L. Feng W. (Springer: Berlin Heidelberg) (2006). 4222:264–73 10.1007/11881223_33

[16] van den Heuvel MP Stam CJ Boersma M Hulshoff Pol HE Small-world and scale-free organization of voxel-based resting-state functional connectivity in the human brain. Neuroimage (2008) 43:528–39 10.1016/j.neuroimage.2008.08.010 18786642

[17] Stam CJ van Straaten EC The organization of physiological brain networks. Clin Neurophysiol (2012) 123(6):1067–87 10.1016/j.clinph.2012.01.011 22356937

[18] Bernhardt BC Chen Z He Y Evans AC Bernasconi N Graph-theoretical analysis reveals disrupted small-world organization of cortical thickness correlation networks in temporal lobe epilepsy. Cereb Cortex (2011) 21:2147–57 10.1093/cercor/bhq291 21330467

[19] Netoff TI Clewley R Arno S Keck T White JA Epilepsy in small-world networks. J Neurosci (2004) 24:8075–83 10.1523/JNEUROSCI.1509-04.2004 15371508 PMC6729784

[20] Hsu T-W Wu CW Cheng Y-F Chen H-L Lu C-H Cho K-H Impaired small-world network efficiency and dynamic functional distribution in patients with cirrhosis. PLoS One (2012) 7:e35266 10.1371/journal.pone.0035266 22563460 PMC3341390

[21] Liang Z King J Zhang N Intrinsic organization of the anesthetized brain. J Neurosci (2012) 32:10183–91 10.1523/JNEUROSCI.1020-12.2012 22836253 PMC3422560

[22] Schröter MS Spoormaker VI Schorer A Wohlschläger A Czisch M Kochs EF Spatiotemporal reconfiguration of large-scale brain functional networks during propofol-induced loss of consciousness. J Neurosci (2012) 32:12832–40 10.1523/JNEUROSCI.6046-11.2012 22973006 PMC6703804

[23] Jann K Dierks T Boesch C Kottlow M Strik W Koenig T BOLD correlates of EEG alpha phase-locking and the fMRI default mode network. Neuroimage (2009) 45:903–16 10.1016/j.neuroimage.2009.01.001 19280706

[24] Fell J Axmacher N The role of phase synchronization in memory processes. Nat Rev Neurosci (2011) 12:105–18 10.1038/nrn2979 21248789

[25] Cummins RO Chamberlain DA Abramson NS Allen M Baskett PJ Becker L Recommended guidelines for uniform reporting of data from out-of-hospital cardiac arrest: the Utstein Style. A statement for health professionals from a task force of the American Heart Association, the European Resuscitation Council, the Heart and Stroke Foundation of Canada, and the Australian Resuscitation Council. Circulation (1991) 84(2):960–75 1860248 10.1161/01.cir.84.2.960

[26] Rubinov M Sporns O Complex network measures of brain connectivity: uses and interpretations. Neuroimage (2010) 52:1059–69 10.1016/j.neuroimage.2009.10.003 19819337

[27] Eccles RM Loyning Y Oshima T Effects of hypoxia on the monosynaptic reflex pathway in the cat spinal cord. J Neurophysiol (1966) 29:315–31 5927464 10.1152/jn.1966.29.2.315

[28] Hofmeijer J van Putten MJ Ischemic cerebral damage: an appraisal of synaptic failure. Stroke (2012) 43:607–15 10.1161/STROKEAHA.111.632943 22207505

[29] Horner CH Davies HA Stewart MG Hippocampal synaptic density and glutamate immunoreactivity following transient cerebral ischaemia in the chick. Eur J Neurosci (1998) 10:3913–7 10.1046/j.1460-9568.1998.00435.x 9875369

[30] Cervós-Navarro J Diemer NH Selective vulnerability in brain hypoxia. Crit Rev Neurobiol (1991) 6:149–82 1773451

[31] Wang SS-H Shultz JR Burish MJ Harrison KH Hof PR Towns LC Functional trade-offs in white matter axonal scaling. J Neurosci (2008) 28:4047–56 10.1523/JNEUROSCI.5559-05.2008 18400904 PMC2779774

[32] Reimer MM McQueen J Searcy L Scullion G Zonta B Desmazieres A Rapid disruption of axon-glial integrity in response to mild cerebral hypoperfusion. J Neurosci (2011) 31:18185–94 10.1523/JNEUROSCI.4936-11.2011 22159130 PMC4337974

[33] Tononi G Edelman GM Consciousness and complexity. Science (1998) 282:1846–51 10.1126/science.282.5395.1846 9836628

[34] van Putten MJ Proposed link rates in the human brain. J Neurosci Methods (2003) 127:1–10 10.1016/S0165-0270(03)00090-6 12865143

[35] Pedersen GL Rasmussen SB Gyllenborg J Benedek K Lauritzen M Prognostic value of periodic electroencephalographic discharges for neurological patients with profound disturbances of consciousness. Clin Neurophysiol (2013) 124:44–51 10.1016/j.clinph.2012.06.010 22809812

[36] San-Juan D Chiappa KH Cole AJ Propofol and the electroencephalogram. Clin Neurophysiol (2010) 121:998–1006 10.1016/j.clinph.2009.12.016 20071229

[37] Peraza LR Asghar AU Green G Halliday DM Volume conduction effects in brain network inference from electroencephalographic recordings using phase lag index. J Neurosci Methods (2012) 207:189–99 10.1016/j.jneumeth.2012.04.007 22546477

[38] Stam CJ Nolte G Daffertshofer A Phase lag index: assessment of functional connectivity from multi channel EEG and MEG with diminished bias from common sources. Hum Brain Mapp (2007) 28:1178–93 10.1002/hbm.20346 17266107 PMC6871367

[39] Vinck M Oostenveld R van Wingerden M Battaglia F Pennartz CM An improved index of phase-synchronization for electrophysiological data in the presence of volume-conduction, noise and sample- size bias. Neuroimage (2011) 55:1548–65 10.1016/j.neuroimage.2011.01.055 21276857

[40] Nolte G Bai O Wheaton L Mari Z Vorbach S Hallett M Identifying true brain interaction from EEG data using the imaginary part of coherency. Neurophysiol Clin (2004) 115:2292–307 10.1016/j.clinph.2004.04.029 15351371

[41] Fein G Raz J Brown FF Merrin EL Common reference coherence data are confounded by power and phase effects. Electroencephalogr Clin Neurophysiol (1988) 69:581–4 10.1016/0013-4694(88)90171-X 2453336

